# Genetic effect of CysLTR2 polymorphisms on its mRNA synthesis and stabilization

**DOI:** 10.1186/1471-2350-10-106

**Published:** 2009-10-20

**Authors:** Jeong-Ah Shin, Hun Soo Chang, Se-Min Park, An-Soo Jang, Sung Woo Park, Jong Sook Park, Soo-Taek Uh, Gune Il Lim, Taiyoun Rhim, Mi-Kyeong Kim, Inseon S Choi, Il Yup Chung, Byung Lae Park, Hyoung Doo Shin, Choon-Sik Park

**Affiliations:** 1 Genome Research Center for Allergy and Respiratory Diseases, Soonchunhyang University Bucheon Hospital, 1174, Jung Dong, Wonmi Ku, Bucheon, Gyeonggi Do, 420-767, Korea; 2 Pharmacogenomic Research Center for Psychotropic Drugs, Korea University, 126-1, 5-Ga Anam-Dong, Seongbuk-Gu, Seoul 136-705, Korea; 3 Division of Allergy and Respiratory Disease, Soonchunhyang University, Bucheon Hospital, Korea; 4 Division of Allergy and Respiratory Disease, Soonchunhyang University Seoul Hospital, Korea; 5 Division of Allergy and Respiratory Disease, Soonchunhyang University, Gumi Hospital, Korea; 6 Department of Bio Engineering, Hanyang University, 17 Haengdang-dong, Seongdong-gu, Seoul, 133-791, Korea; 7 Department of Internal Medicine, Chungbuk National University, College of Medicine, 62 Gaesin-dong, Heungduk-gu, Cheongju, Chungcheongbuk-do, 361-711, Korea; 8 Department of Allergy, Chonnam National University Medical School and Research Institute of Medical Sciences, 8 Hakdong, Dong-gu, Gwangju, 501-757, Korea; 9 Division of Molecular & Life Science, The College of Science & Technology, Hanyang University, 1271 Sa 1 Dong, Sangrok Ku, Ansan, Gyeonggi Do, 426-791, Korea; 10 Department of Genetic Epidemiology, SNP Genetics, Inc., 14th Floor, Woorim Lions B/D, 371-28 Kasan Dong, Geumcheon Ku, Seoul, 110-834, Korea; 11 Department of Life Science, Sogang University, 1 Shinsu-dong, Mapo-gu, Seoul, 121-742, Korea

## Abstract

**Background:**

We previously demonstrated that single nucleotide polymorphism (SNP) and haplotypes were associated with aspirin hypersensitivity in asthmatics. We investigated the genetic effects of the SNPs and haplotypes on the expression of the *CysLTR2 *gene.

**Methods:**

We measured CysLTR2 protein and mRNA expression in EB virus-infected B cell lines from asthmatics having *ht1*^+/+ ^and *ht2*^+/+^. A gel retardation assay was used to identify nuclear protein binding to the c.-819 promoter site. The function of promoter and 3'-UTR were assessed using pGL3 luciferase and pEGFP reporter system, respectively.

**Results:**

We found that the expression of CysLTR2 protein was higher in B cell lines of asthmatics having *ht2*^+/+ ^than in those having *ht1*^+/+^. PMA/ionomycin induced higher mRNA expression of CysLTR2 in B cell lines from *ht2*^+/+ ^asthmatics than those from *ht1*^+/+ ^asthmatics. A nuclear protein from the B cell lines showed stronger DNA binding affinity with a probe containing *c*.-*819T than *one containing *c*.-*819G*. The luciferase activity of the *c*.-*819T *type of *CysLTR2 *promoter was higher than that of the *c*.-*819G *type. EGFP expression was higher in the EGFP-*c.2078T *3'-UTR fusion construct than in the *c.2078C *construct.

**Conclusion:**

The sequence variants of *CysLTR2 *may affect its transcription and the stability of its mRNA, resulting in altered expression of CysLTR2 protein, which in turn causes some asthmatics to be susceptible to aspirin hypersensitivity.

## Background

Aspirin-intolerant asthma (AIA) refers to the development of bronchoconstriction in asthmatics following the ingestion of aspirin or other non-steroidal anti-inflammatory drugs (NSAIDs). This syndrome is characterized by the 'aspirin triad' [1] syndrome of aspirin hypersensitivity, bronchial asthma, and chronic rhinosinusitis with nasal polyposis. A two-compartment model has been proposed in which both the augmentation of cysteinyl leukotriene (CysLT) production and the overexpression of the CysLT receptor on inflammatory cells occur within the respiratory tract [2]. The overproduction of CysLTs has been demonstrated in the airways and circulation of asthmatics who are intolerant to aspirin [3-5]. CysLTs are important inflammatory mediators in the development of asthma, as they mediate bronchoconstriction while increasing mucus secretion, vascular permeability and cellular infiltration [6]. CysLTs exert their biological actions by binding two types of G-protein-coupled seven-transmembrane receptors: cysteinyl leukotriene receptor 1 (CysLTR1; MIM 300201), which is sensitive to the asthma drugs montelukast, zafirlukast and pranlukast [7,8] and CysLTR2 [9]. CysLTR2 has been documented to be expressed in lung interstitial macrophage [10], pulmonary vascular smooth muscle, endothelium [11-13], eosinophils [14], mast cells [15], B and T lymphocytes [16,17], raising the possibility that CysLTR2 may have an important role in allergic inflammation. Particularly, CysLTR2 may have important role in remodeling and fibrosis pathways in B lymphocytes. Furthermore, CysLTR2 is located on chromosome13q14.2-21.1, near a locus known to be associated with the risk of asthma in various population. CysLTR2 play a disease-regulating role in glands and epithelium of rhinosinusitis, particularly aspirin-sensitive disease [18]. In addition, Cysteinyl-LT signaling may provide a key balance between a release of endothelium-dependent relaxant and constricting factors in endothelium dysfunction [19]. The endothelial CysLTR2 is rapidly down-regulated by pro-inflammatory stimuli in Myocardial Ischemia [20]. A role for CysLTR2 is mediated the coronary constriction in patients with coronary artery disease [21]. CysLTR2 may also contribute to neurological inflammation in the human brain and the adrenal glands of neuroendocrine system [22]. We previously identified four sequence variants of the *CysLTR2 *gene: one in the promoter (*c*.-*819G>T, rs7324991*), two in the 3'-flanking region (*c.2078C>T *and *c.2534A>G, novel and rs912278*), and one downstream of the gene (*c.2545+297A>G, rs2407249*) [20]. We also revealed that asthmatics having rare alleles for *c*.-*819G>T*, *c.2078C>T *or *c.2534A>G *exhibited a more pronounced drop in FEV_1 _by aspirin provocation than those who carried the common alleles (*P *= 0.03-0.009). In previously study, five haplotypes (GCAA, TTGA, TCGG, TCAA and others) were constructed. The frequencies of these five haplotypes in the Korean population (N = 642), African-American (N = 50) and Caucasian (N = 50) were: 0.495, 0.14, 0.44 (*ht1*), 0.346, 0.02, 0.23 (*ht*2), 0.083, 0.17, 0.23 (*ht3*), 0.07, 0.6, 0.09 (*ht4*) and 0.006, 0.07, 0.01 (*others*) in Korean, African-American and Caucasian, respectively. Significant differences in the frequencies of the SNPs and haplotypes were observed among the three ethnic groups. The linkage disequilibrium coefficients (|D'|) and *r*^2 ^among the SNPs were calculated for all of the study subjects. Strong LDs were noted between SNPs (|D'| > 0.97) in Korean. Complete LDs were observed between SNPs (c.-819 G>T, c.2078 C>T and c.2534 A>G) in Caucasian (|D'| > 0.90). In African-American, between SNPs (c.2534 A>G and c.2545+297A>G) were observed complete LDs (|D'| > 0.92) [20]. Subjects with the ht2 haplotype, which is composed of the rare alleles for the three SNPs (*TTG*), were susceptible to aspirin intolerance. These results suggest that sequence variants on the promoter (*c*.-*819G>T*) of *CysLTR2 *may affect the efficiency of its transcription with tight linkage to 3'-UTR variations or that the SNPs themselves on 3'-UTR may affect the stability of its mRNA [20]. The functional effects of these SNPs on transcription should be identified. Here, we investigated the genetic effect of the promoter and the 3'-UTR polymorphisms of CysLTR2 at the transcriptional and translational levels of the gene.

## Results

### Flow cytometric analysis of CysLTR2 expression between haplotypes 1 and 2

**Figure 1:**
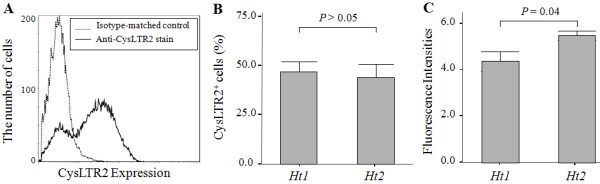
**Flow cytometric analysis of CysLTR2 expression on B cell lines between haplotype 1 homozygote (*ht1*^+/+^, *GCAA*: *c*.--*819G>T*, *c.2078C>T*, *c.2534A>G *and c.*2545+297A>G*) and haplotype 2 homozygote (*ht2*^+/+^, *TTGG*)**. (A) Histogram of CysLTR2 protein expression on the EBV-infected B cell line. Immunofluorescence staining was performed using anti-human CysLTR2 Ab and FITC-conjugated secondary Ab as described in the *Methods*. The histogram is a representative of 17 independent experiments. (B) Ratio of CysLTR2 positive cells of the EBV-infected B cell lines having *ht1*^+/+ ^(*n *= 8) or *ht2*^+/+ ^(*n *= 9). (C) Mean fluorescence intensity of CysLTR2 expression of the same B cell lines. The ratio of mean FL1 channel values was determined as the FL1 level of CysLTR2 staining to that of isotype-matched control staining. *P *values were calculated by a Mann--Whitney *U*-test.

To investigate the effect of the SNPs on the production of CysLTR2 protein in the B cell lines, we measured CysLTR2 protein levels on the surface of B cell lines for ht1 homozygotes (*ht1*^+/+^, N = 8) and ht2 homozygotes (*ht2*^+/+^, N = 9) using immunofluorescence staining and flow cytometric analysis (Figure 1). Approximately 50% of EBV-infected B cells expressed CysLTR2 on their surface (Figure 1A). The positive rate of CysLTR2 on the B cell lines was not significantly different between the two haplotypes (*ht1*^+/+^; 46.96 ± 5.10% vs. *ht2*^+/+^; 48.26 ± 7.36%, *P *> 0.05; Figure 1B). The amount of CysLTR2 protein among CysLTR2 positive B cells, however, was significantly different (Figure 1C). The mean fluorescence intensity was significantly higher in B cells of the subjects having *ht2*^+/+ ^than in those having *ht1*^+/+ ^(5.07 ± 0.21 vs. 3.90 ± 0.45, *P *= 0.04). These results indicate that single nucleotide polymorphisms on the promoter or 3'-UTR region may affect protein expression of the *CysLTR2 *gene.

### Comparison of mRNA levels of the *CysLTR2 *gene between haplotypes 1 and 2

**Figure 2:**
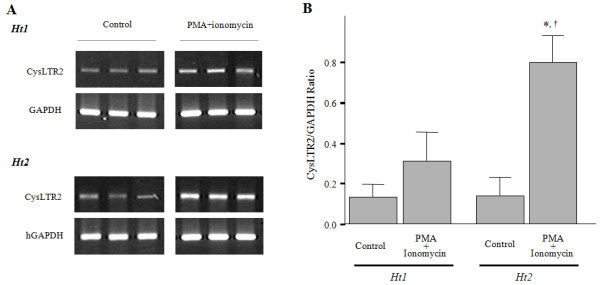
**CysLTR2 mRNA expression of *ht1*^+/+ ^or *ht2*^+/+ ^EBV-infected B cell lines**. After B cell lines were cultured with or without PMA (10 ng/ml)/ionomycin (2 μM) for 24 h, the mRNA expression of CysLTR2 was analyzed by RT--PCR. (A) The intensities of PCR products were measured using a densitometer, and compared between *ht1*^+/+^(*n *= 6) and *ht2*^+/+ ^(*n *= 6) B cell lines. (B) The data are presented as the mean ± SEM. * *P *< 0.05 calculated using a Mann--Whitney *U*-test for comparing PMA/ionomycin-treated *ht1*^+/+ ^or *ht2*^+/+ ^B cell lines. † *P *< 0.01 calculated using Wilcoxon's signed-ranks test for comparing PMA/ionomycin-treated and untreated *ht2*^+/+ ^B cell lines.

To investigate the genetic effect of these polymorphisms on the mRNA expression of *CysLTR2 *in B cell lines, we measured the CysLTR2 mRNA levels between *ht1*^+/+^and *ht2*^+/+ ^by RT-PCR. Semi-quantitation of CysLTR2 mRNA expression was measured by the intensity of CysLTR2 RT-PCR product corrected by the intensity of GAPDH. The expression level of CysLTR2 mRNA in unstimulated B cell lines was similar in *ht2*^+/+ ^(N = 6) and *ht1*^+/+ ^(N = 6, *P >*0.05; Figure 2A); however, PMA/ionomycin markedly induced mRNA expression of the *CysLTR2 *gene in EBV-infected B cells of *ht2*^+/+ ^(N = 6) relative to *ht1*^+/+ ^(N = 6, *P *= 0.016; Figure 2B). These results indicate that the protein expression of the *CysLTR2 *gene may be regulated at transcriptional levels.

### Comparison of promoter activity between T and G type of *CysLTR2 *promoter *c.-819 T>G*

Because the SNPs of *CysLTR2 *haplotype 1 and 2 are located on the promoter region and on the 3'-UTR region, we investigated the individual effect of these two SNPs on CysLTR2 mRNA expression. First, we compared the activities of promoters possessing *c*.-*819T *or *G *by luciferase reporter assay as described in the *Methods*.

**Figure 3:**
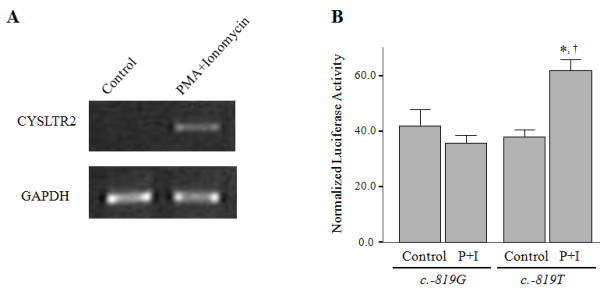
**Promoter (*c*.--*819T>G*) activity of the human *CysLTR2 *gene**. The 293 T cells expressed CysLTR2, which increased with 24 h PMA/ionomycin stimulation (A) The promoter region of *CysLTR2 *having c.--819T or c.--819G was inserted into the pGL3 basic vector and the constructs and β-galactosidase expression vector were co-transfected into 293 T cells by lipofection. Twenty-four hours after transfection, the transfected cells (2.5 × 10^5^/ml) were stimulated with 10 ng/ml of PMA and 2 mM of ionomycin for 24 h. Luciferase activities were measure by luminometer and normalized by β-galactosidase activities as internal controls. (B) The data are presented as the mean ± SEM of normalized luciferase activities (the ratio of luciferase activities to β-galactosidase activities) of six independent experiments. *P < 0.01 calculated using a Mann--Whitney *U*-test for comparing luciferase activities between PMA/ionomycin-treated *c*.--*819G *and *c*.--*819T *promoter. † *P *< 0.05 calculated using Wilcoxon's signed-ranks test for comparing luciferase activities of PMA/ionomycin-treated and untreated *c.819T *promoter.

Due to 293 T cells expressing CysLTR2 mRNA by PMA/ionomycin stimulation (Figure 3A), the *CysLTR2 *promoter-luciferase constructs were transfected into the cells and the differences in luciferase activities between *c*.-*819G *and *T *were analyzed. Luciferase activity was normalized by β-galactosidase activity co-transfected as an internal control. In the absence of stimulation, no difference was observed in luciferase activities between genotypes (Figure 3B, *c*.-*819G*; 41.83 ± 6.01 vs. *c*.-*819T*; 38.12 ± 2.28, *P *> 0.05). However, PMA/ionomycin stimulation significantly induced luciferase activity in *c*.-*819T *but not in the *G *type. As a result, the luciferase activity of the *c*.-*819T *type of the *CysLTR2 *promoter was significantly higher than that of the *c*.-*819G *type (60.79 ± 4.21 vs. 35.30 ± 2.70, respectively, *P *= 0.002). These data suggest that transcription of the *CysLTR2 *gene may be differently regulated when stimulated according to the nucleotide polymorphism at the -*819 *locus on the promoter.

### Nuclear factor binding site on the region of the promoter containing *CysLTR2 c.-819T>G*

**Figure 4:**
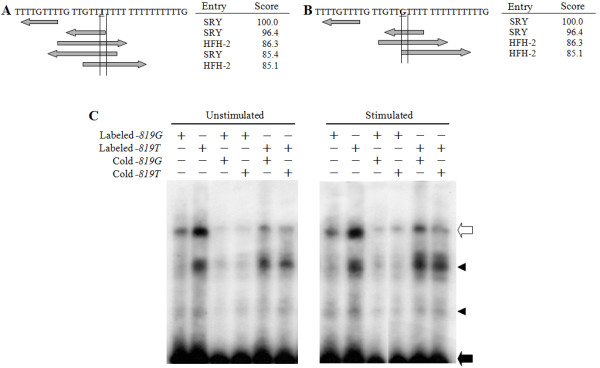
**Gel shift assay for SNP *CysLTR2 c*.--*819 *using nuclear extract from B cell lines**. Putative transcription factor binding site of *c.--819T *(A) and *G *type (B) are indicated by underlines (TFSEARCH Searching Transcriptional Factor Binding Sites V1.3). Gel shift assays were performed using nuclear extracts of EBV-infected B cell lines and dsDNA probes possessing *c.--819G *or *T *(C). One million cells per milliliter of EBV-infected B cell lines established from asthmatics were cultured for 24 h with or without 10 ng/ml of PMA and 2 μM of ionomycin. The nuclear extracts of B cell lines were incubated with the ^32^P-labeled probes, the sequences of which included each genotype of c.--819 (see *Methods*). To identify specific binding complexes, nuclear extract from B cell lines was preincubated with the unlabeled probe 10 min before the addition of the labeled probe. The open arrow shows the specific DNA--protein complexes and the filled arrow indicates the free unbound probe. Arrowheads denote nonspecific DNA--protein complexes. These data are representative of six independent experiments.

To confirm whether the locus on the promoter of *CysLTR2 *(*c*.-*819G>T) provided the *binding sites for a transcription factor, gel shift assays were performed using the nuclear extract from EBV-infected B cells. DNA-protein complexes were detected with the probe for *CysLTR2 c*.-*819T *(Figure 4C). The intensity of this complex was higher when hybridized with the probe for *c*.-*819T than with the probe for *the *c*.-*819G and *decreased through competition with the unlabeled probe of each type. Nuclear extract from the PMA/ionomycin-stimulated B cell lines showed stronger DNA-protein binding affinity in the T type of probe than the unstimulated B cell lines. This indicates that the *CysLTR2 promoter region around c*.-*819T may be the binding site for a transcription *factor. Since a TFSEARCH search revealed putative binding sites for several candidate transcription factors around the *c*.-*819T>G *allele, such as HFH-2 and SRY (Figure 4A and 4B), we performed a supershift assay for SRY and Foxd3, which is the human homolog of HFH-2. However, neither HFH-2 nor SRY showed the supershift in EMSA (data not shown).

### Comparison of 3'-UTR activity between C and T types of the *CysLTR2 *3'- UTR *c.2078C>T*

**Figure 5:**
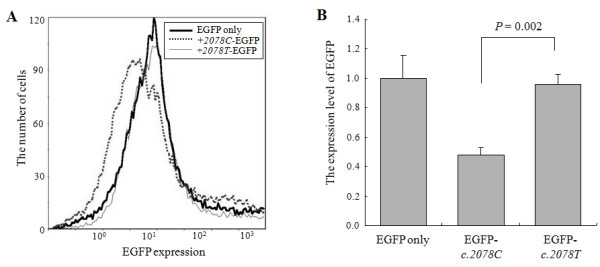
**Flow cytometric analysis of the effects of the human *CysLTR2 *gene 3'-UTR on EGFP protein expression**. The 3'-UTR region possessing each genotype (*C *or *T*) on c.2078 was inserted between the EGFP coding region and SV40 poly A site as described in the *Methods*. After each construct was co-transfected with the β-galactosidase expression vector as an internal control into 293 T cell lines, the expression levels of the EGFP protein were measured using a flow cytometer (A) and the fluorescence intensity of each construct was compared (B). The data are presented as the mean ± SEM of relative FL1 values (the ratio of EGFP--CysLTR2 3'-UTR fusion constructs to naive pEGFP-N1 vector) of six independent experiments. The *P *value was calculated using a Mann--Whitney *U*-test.

To evaluate the effect due to sequence variance of 3'-UTR *c.2078C>T *on CysLTR2 protein expression, the fluorescence intensity of 293 T cell lines transfected with each type (*c.2078C *or *T) *of EGFP-*CysLTR2 *3'-UTR fusion construct was analyzed using flow cytometry (Figure 5A). The EGFP expression levels of the EGFP-*CysLTR2 *3'-UTR fusion constructs were analyzed as the mean fluorescence intensity of EGFP of all cells analyzed. On flow cytometry analysis, side scatter and forward scatter were not different between the two cell types. The relative EGFP expression level of the 3'-UTR (*c.2078T) *fusion construct was significantly higher than that of the *c.2078C *construct (Figure 5B; 0.96 ± 0.07 vs. 0.48 ± 0.06, respectively, *P *= 0.002), indicating that the mRNA stability of the *c.2078T *type of CysLTR2 3'-UTR was higher than that of *c.2078C*.

## Discussion

We demonstrated that the sequence variant on the promoter (*c*.-*819G>T*) of the *CysLTR2 *gene affects the efficiency of its transcription and the sequence variant on the 3'-UTR (*c.2078C>T) *modulates mRNA degradation, resulting in different expressions of *CysLTR2 *protein according to the gene genotypes. We previously identified three novel SNPs in the promoter and 3'-untranslated region of CysLTR2. Three SNPs (*c*.-*819G>T*, *c.2078C>T*, and *c.2534A>G*) and *ht1 *[*G*-*C*-*A*-*A*], *ht2 *[*T*-*T*-*G*-*A*] were strongly associated with the drop in FEV_1 _induced by aspirin challenge in asthmatics [20]. To identify the functional effects of these SNPs on transcription and translation of the *CysLTR2 *gene, we first measured the protein expression of the *CysLTR2 *gene. CysLTR2 is known to be expressed by lung interstitial macrophages [10], pulmonary vascular smooth muscle, endothelium [11-13], eosinophils [14] and mast cells [15]. These cells are difficult to obtain and maintain from our asthmatics subjects, so we used immortalized B cells induced by EB virus from subjects whose genotypes had been analyzed in a previous study [20]. We demonstrated that the B cell lines having *ht2*^+/+ ^had a higher translational and transcriptional expression levels of CysLTR2 than those having *ht1*^+/+^, especially when stimulated (Figure 1 and 2). These results indicate that the genetic differences affect the amount of protein and mRNA expression in the *CysLTR2 *gene. In our previous studies, *ht2 *homozygote asthmatics showed the highest responses to aspirin challenge, while *ht1 *homozygotes showed the lowest responses [20]. Taken together, these data suggest that the greater response to aspirin challenge in asthmatics may be due to higher expression of CysLTR2 protein on the B cells and presumably on the other *CysLTR2 *gene-expressing cells, such as lung interstitial macrophages [10], eosinophils [14] and mast cells [15].

Next, we assessed the regulating mechanisms of the three SNPs (*c*.-*819G>T*, *c.2078C>T *and *c.2534A>G*) on CysLTR2 protein expression using promoter activity assays and 3'-UTR function analysis because the sequence variants on the promoter (*c*.-*819G>T*) of the *CysLTR2 *gene may affect the efficiency of its transcription and the SNPs on the 3'-UTR may affect the stability of its mRNA. Luciferase activity of the *CysLTR2 *promoter was higher in the *T *type transfected 293T cells than in the *G *type, which indicates that the sequence variants on the promoter are regulators for *CysLTR2 *gene expression (Figure 3). The genetic role of the promoter (*c*.-*819G>T*) was demonstrated by the nuclear protein binding assay (Figure 4). The binding affinity of this DNA-binding factor was higher for *c*.-*819T than for c*.-*819G *with or without PMA/ionophore stimulation (Figure 4). In the steady state, this DNA-binding factor may bind constitutively to the promoter close to the *c*.-*819G>T locus and *induce *CysLTR2 *gene expression. Moreover, PMA/ionophore-stimulated signaling may cause an increase in this binding factor on the promoter, which would induce greater *CysLTR2 gene expression. With c.-819T*, PMA/ionophore-induced signaling may cause higher-level binding of the DNA-binding factor than it does with *c*.-*819G, which would *increase *CysLTR2 gene expression. We performed a *supershift assay for the candidate factors: SRY and HFH-2. Neither SRY nor FoxD3 (human homolog of HFH2) antibodies showed supershifts in EMSA. The nature of the putative DNA-binding factor therefore remains to be clarified.

In the evaluation of the sequence variance of the 3'-UTR (*c.2078C>T *and *c.2534A>G*) on *CysLTR2 *gene expression, the former was chosen since the *c.2534A>G *is located 124 bp downstream from the polyadenylation site and would not be present in mature mRNA. The expression level of fluorescence containing the *c.2078T *type was significantly higher than that of *c.2078C *(Figure 5). This indicates that mRNA degradation of the T type of *CysLTR2 *3'-UTR was much slower than that of the C type. Based on the results of our promoter and 3'-UTR functional study, the sequence variance of the two loci may influence *CysLTR2 gene expression *cooperatively. To validate the results of our promoter and 3'-UTR functional study, we performed the additional experiment using the vectors containing both promoter and 3'-UTR regions of CysLTR2. *Ht1 *(c. -819 G and c. 2078 C) and *Ht2 *(c. -819 T and c. 2078 T) were inserted into pGL3-basic luciferase reporter system, respectively. The expression level of luciferase containing the *ht2 *was significantly higher than that of *ht1*, especially when stimulated with PMA plus ionomycin (data not shown). These results indicate that the combined genetic differences affect the amount of protein and mRNA expression in CysLTR2 gene. In contrast to the extensive studies on the functions of the CysLT_1 _receptor [21,22], the functional characterization of the CysLT_2 _receptor has been limited because of a lack of specific competitive antagonists. Recent experimental studies have demonstrated unanticipated functions for the cysLTs, acting nonredundantly through CysLTR2. CysLTR2, but not CystLTR1, is the receptor responsible for mediating the contribution of the CysLTs to experimental bleomycin-induced chronic pulmonary inflammation and fibrosis [23], and for the production of IL-8 by IL-4-primed cultured human mast cells [24]. However, the impact of the receptor has not been revealed in chronic inflammatory airway diseases such as asthma. In particular, any *in vivo *role for the CysLTR2 receptor remains unknown. To the best of our knowledge, our study is the first to clearly demonstrate the genetic relationship between the CysLTR2 receptor nucleotide polymorphism and aspirin hypersensitivity in asthmatics at the level of gene function.

## Conclusion

The sequence variants on the promoter and on the 3'-UTR of *CysLTR2 *affect the efficiency of its transcription and the stability of its mRNA, resulting in the alteration of protein expression of CysLTR2. This change causes a certain number of asthmatics to be susceptible to aspirin hypersensitity. This functional evidence may have genetic value as a diagnostic biomarker and could provide fundamental information for the development of therapeutic strategies.

## Methods

### EV virus-infected B cell culture

Venous whole blood was obtained from asthmatics having the haplotype 1 (common allele homozygotes (*GCAA*: *c*.-*819G>T*, *c.2078C>T, c.2534A>G *and c.*2545+297A>G*) and haplotype 2 (rare allele homozygotes (*TTGG*)) and peripheral blood mononuclear cells (PBMC) were separated using discontinuous Histopaque gradients solution (Sigma, St. Louis, MO). PBMC were infected with B95-8 supernatant and incubated with 0.5 μg/ml of cyclosporine A (Sigma) for 3 weeks. Cells were cultured in RPMI 1640 (Invitrogen Life Technologies, Carlsbad, CA) supplemented with L-glutamine (2 mM), HEPES (25 mM), and 10% FBS (Invitrogen Life Technologies). All cell lines were maintained in culture at 37°C in an atmosphere containing 5% CO_2_. Cells were reseeded at a density of 5 × 10^4 ^cells/ml every 3-4 days. This study was performed with the approval of the Ethics Committee of the Soonchunhyang University Hospital and informed written consent was obtained from all the study subjects.

### Flow cytometry analysis of CysLTR2 protein expression

The expression of CysLTR2 in B cell lines was assessed with the use of a polyclonal anti-CysLTR2 antibody (Cayman Chemicals, Ann Arbor, MI) directed against amino acids 1-18 of the N-terminal portion of the receptor. For flow cytometry studies, B cell lines were washed twice with PBS and labeled for 30 min at 4°C with anti-CysLTR2 antibody (or irrelevant antibody for control). Cells were then washed twice with PBS and incubated for 30 min at 4°C with FITC-conjugated goat anti-rabbit IgG (BD Pharmingen™; BD Biosciences, San Diego, CA). As an isotype-matched control, FITC-conjugated mouse IgG1κ (BD Biosciences) was similarly treated. Finally, cells were washed again and resuspended in PBS. An analysis of fluorescence staining was performed with a FACSCalibur flow cytometer and Cell Quest Software (Counter Corporation, Miami, FL). The levels of CysLTR2 expression were determined as the percentage of positive staining and the ratio of mean FL1 channel values of CysLTR2 stain. The percentage of positive staining was measured as the cells showing fluorescence above that of the control antibody. The ratio of mean FL1 channel values was determined as the FL1 level of CysLTR2 stain to that of the isotype-matched control stain.

### Semi-quantitation of mRNA expression using reverse transcriptase polymerase chain reaction

Total RNA was extracted using TRI Reagent (guanidium thiocyanate-phenol mixture; Molecular Research Center, Inc., Cincinnati, OH) and chloroform. Possible DNA contamination was eliminated by treatment with 1 μl of RNase-free DNase I (10,000 U/ml; Stratagene, La Jolla, CA) for 15 min at 37°C. The reaction was stopped with 0.5 μl of 2 mM EDTA. DNase I-treated RNA was heated at 65°C for 5 min with 1 μl of oligo deoxythymidine-15 primer (500 μg/ml) and 10 mM dNTP mix, then quickly chilled on ice. After centrifugation, DNase I-treated RNA were incubated at 42°C for 2 min with 0.1 mM DTT and 1 μl of SuperScript II™ RT (200 U/μl; Invitrogen Life Technologies) at 42°C for 50 min, and then heat inactivated at 70°C for 15 min. Following reverse transcription (RT), cDNA was aliquoted into tubes containing specific primer pairs (Table 1) for human CysLTR2 and GAPDH genes to amplify into 706-bp and 300-bp PCR products, respectively. Amplification was performed on a thermocycler for 30 cycles (one cycle: 1 min at 95°C, 1 min at 55°C, and 1 min at 72°C) with the initial denaturation at 95°C for 5 min and a final extension at 72°C for 7 min. Amplified PCR products were electrophoresed on a 1% agarose gel and visualized by ethidium bromide staining.

**Table 1:** Primer sequences used in this study

**Approach**	**Orientation**	**Location**	**Primer Sequence**
RT-PCR			
CysLTR2	Forward	+29/+46	5'-CATCCATCTCCGTATCAG-3'
	Reverse	+734/+717	5'-GCCTTCCTGTGAGAAACC-3'
GAPDH	Forward		5'-CGTCTTCACCATGGAGA-3'
	Reverse		5'-CGGCCATCACGCCACAGTTT-3'
Promoter construct	Forward	-1342/-1323	5'-TTTTCCTGCCTTGTTGTTGG-3'
	Reverse	+178/+159	5'-TGGACAACCCATTTCCCAAG-3'
	Nested, Forward	-1008/-987	5'-ATTTAGCTAGCCAAAACATTAAATGTAACTTAG-3'
	Nested, Reverse	-4/-30	5'-ATTATCTCGAGGGGTTAAAAAGAAACAGACACAAAAAG-3'
UTR construct	Forward	+501/+517	5'-GGCTTCCTCAATAATGC-3'
	Reverse	+2787/+2770	5'-GGTTGACCAAATGCTGTG-3'
	Nested, Forward	+1042/+1062	5'-AAATAGCGGCCGCGGAGCTCTTAGATGAGACCTG-3'
	Nested, Reverse	+2410/+2385	5'-TTAATGCGGCCGCTTGGTAGGAAAGGACAGCTTTTATTC-3'

Underlined nucleotide sequences are added to the 5' end of oligonucleotide primer for cloning. These cloning sites are *Nhe I *(GCTAGC), *Xho I *(CTCGAG), and Not I (GCGGCCGC), and they are preceded by A and T residues at random

### Preparation of the promoter (*c.-819T>G*)-luciferase and the EGFP-3'-UTR (*c.2078C>T*) reporter constructs

The promoter and UTR region of *CysLTR2 *were amplified by PCR. Primary PCR products were amplified using the genomic DNA of allele-matched B cell lines as templates from the *CysLTR2 *promoter (1520-bp fragment, from -1342 to +178) and UTR (2287 bp fragment, from +501 to +2787) sequence. The primary PCR reaction mixture was diluted and used as a template for a nested PCR using the nested primer. The secondary PCR primer for the promoter (1027-bp fragments, from -1008 to -4) and UTR (1395-bp fragments, from +1042 to +2410) region contained a *NheI*-*XhoI *and *NotI*-*NotI *restriction site. The primers used are listed in Table 1. The amplified PCR product was cloned into a pGEM-T easy vector (Promega, Madison, WI) and digested with *NheI *and *XhoI *(New England Biolabs, Beverly, MA). The fragment was subcloned into the *NheI *and *XhoI *sites of a promoterless luciferase reporter pGL3-Basic vector (Promega). The UTR fragment was cloned into the *NotI *site of an enhanced green fluorescent protein (EGFP) expression plasmid, pEGFP-N1 vector (BD Biosciences). Plasmids were isolated using the Qiagen plasmid purification kit (Qiagen, Santa Clarita, CA).

### Transient transfection and assays of luciferase and fluorescence intensity

The 293 T cells were grown in Dulbecco's modified Eagle's medium (Life Technologies, Rockville, MD) with L-glutamine (2 mM), HEPES (25 mM), 10% heat-inactivated FBS (Invitrogen Life Technologies), 100 U/ml penicillin, and 100 ng/ml streptomycin (Life Technologies-BRL, Gaithersburg, MD) at 37°C in humidified air containing 5% CO_2_. One day before transfection, 5 × 10^5 ^cells were seeded per well in 2 ml without antibiotics to obtain 90-95% confluence at the time of transfection. The 293 T cells were then transiently transfected with pGL3-promoter or pEGFP-UTR of the *CysLTR2 *gene using LF2000 (Invitrogen Life Technologies; recommended μg DNA: μl lipofectamine = 1:2) in OptMEM (Life Technologies-BRL) without serum. One microgram of pSV-β-galactosidase control vector (Promega) was co-transfected to normalize transfection efficiencies. For promoter construct transfection, PMA (10 ng/ml) and ionomycin (2 μM) was added for 24 h after transfection. After 48 h of transfection, cultured cells were washed twice with PBS and solubilized by scraping with 400 μl of reporter lysis buffer (Promega). Cells were centrifuged and the supernatant was stored at -70°C. β-galactosidase activities were assessed by ortho-nitrophenyl-D-galactopyranoside (ONPG) hydrolysis using a β-Galactosidase Enzyme Assay System Kit (Promega) and total protein was measured using a BCA protein assay kit (Pierce, Rockford, IL). Luciferase activity was measured using the Luciferase Assay System (Promega) by a Turner 20/20 n luminometer (Turner BioSystems, Inc., Sunnyvale, CA) and expressed as relative luciferase activity to that of empty pGL3-basic vector. The expression levels of EGFP with the 3'-UTR of *CysLTR2 *were assessed by measuring the mean channel values of FL1 in flow cytometry (BD Biosciences) at 48 h after transfections. The relative mean value of FL1 was calculated as described above. The ratio of mean FL1 channel values was determined as the FL1 level of the transfectant stain to that of the isotype-matched control stain.

### Nuclear extract preparation and electrophoretic mobility shift assays (EMSA)

Nuclear extracts were prepared by the modified method of Schreiber *et al*. [25]. The PMA and ionomycin stimulated B cell lines and unstimulated B cell lines were harvested after washing twice with HBSS. Cells were washed with buffer A (10 mM HEPES, 15 mM KCl, 2 mM MgCl_2_, 0.1 mM EDTA, 1 mM DTT, 0.5 mM PMSF, 10 μg/ml leupeptin, 10 μg/ml aprotinin; Calbiochem, La Jolla, CA). The cell pellet was resuspended in buffer B (buffer A containing 0.2% Nonidet P-40) and incubated for 10 min. Nuclei were pelleted by centrifugation and resuspended in buffer C (buffer A containing 0.25 M sucrose). Nuclei were again pelleted, then resuspended in buffer D (50 mM HEPES, 400 mM KCl, 10% glycerol, 0.1 mM EDTA, 1 mM DTT, 0.5 mM PMSF, 10 μg/ml leupeptin, 10 μg/ml aprotinin; Calbiochem) and incubated with shaking for 30 min. All procedures were performed on ice. The mixture was centrifuged, and the supernatant was stored at -70°C. DNA-protein binding assays were performed using the Gel Shift Assay System (Promega) following the manufacturer's instructions. Ten-microgram aliquots of nuclear extracts were incubated in 10 μl of total reaction volume containing binding buffer (Promega) for 10 min with or without unlabeled oligonucleotide probe. The ^32^P-labeled oligonucleotide probe was added to the reaction mixture and then incubated for 20 min. The reaction products were analyzed by electrophoresis in a 4% polyacrylamide gel with 0.5× TBE buffer. Gels were dried and analyzed by autoradiography. The sequences of the oligonucleotide probes used were forward: 5'-TTTTG TTTTG TTGTT [G/T]TTTT TTTTT TTTTT G-3', reverse: 5-CAAAA AAAAA AAAAA [C/A]AACA ACAAA ACAAA A-3. To determine the specificities of the DNA-protein complexes, 1.75 pmol of unlabeled probe was incubated with the nuclear extract for 10 min before the addition of the labeled probe. For the supershift assay, 200 μg of goat anti-human SRY Ab (Santa Cruz Biotechnology, Santa Cruz, CA) or goat anti-human FoxD3 Ab (Santa Cruz Biotechnology) was incubated with the nuclear extract for 30 min before the addition of the labeled probe.

### Statistical analysis

Data are expressed as the mean ± standard error of the mean (SEM). The program SPSS (version 10.0; SPSS Inc., Chicago, IL) was used for all analyses. Differences between independent groups and samples were compared using the nonparametric Kruskal-Wallis *H*-test for continuous data. If significant differences were found, a Mann-Whitney *U*-test was applied to compare differences between two samples. Wilcoxon's signed-ranks test was used for two related samples.

## Abbreviations

AIA: Aspirin-intolerant asthma; NSAID: non-steroidal anti-inflammatory drugs; CysLT: cysteinyl leukotriene; CysLTR: cysteinyl leukotriene receptor; FEV_1_: Forced expiratory volume in one second; SNP: Single nucleotide polymorphism; LD: Linkage disequilibrium; PBMC: peripheral blood mononuclear cells; EBV: Epstein-Barr virus; EGFP: Enhanced green fluorescent protein; EMSA: Electrophoretic mobility shift assays; SEM: Standard error of the mean.

## Competing interests

The authors declare that they have no competing interests.

## Authors' contributions

JAS performed all the experimental steps, HSC provided experimental assistance, SMP drafted the manuscript, CSP provided analytic support and supervised the project. ASJ, SWP, JSP, STU, GIL, TR, MKK, ISC and IYC provided analytic and data assistance. BLP and HDS supervised the SNP analysis. All authors have read and approved this final manuscript.

## Pre-publication history

The pre-publication history for this paper can be accessed here:



## References

[B1] Samter M, Beers RF (1967). Concerning the nature of intolerance to aspirin. J Allergy.

[B2] Sousa AR, Parikh A, Scadding G, Corrigan CJ, Lee TH (2002). Leukotriene-receptor expression on nasal mucosal inflammatory cells in aspirin-sensitive rhinosinusitis. N Engl J Med.

[B3] Picado C (2002). Aspirin-intolerant asthma: role of cyclo-oxygenase enzymes. Allergy.

[B4] Szczeklik A, Gryglewski RJ, Czerniawska-Mysik G (1975). Relationship of inhibition of prostaglandin biosynthesis by analgesics to asthma attacks in aspirin-sensitive patients. Br Med J.

[B5] Szczeklik A (1990). The cyclooxygenase theory of aspirin-induced asthma. Eur Respir J.

[B6] Henderson WR (1994). The role of leukotrienes in inflammation. Ann Intern Med.

[B7] Drazen JM, Israel E, O'Byrne PM (1999). Treatment of asthma with drugs modifying the leukotriene pathway. N Engl J Med.

[B8] Leff AR (2001). Regulation of leukotrienes in the management of asthma: biology and clinical therapy. Annu Rev Med.

[B9] Hui Y, Funk CD (2002). Cysteinyl leukotriene receptors. Biochem Pharmacol.

[B10] Heise CE, O'Dowd BF, Figueroa DJ, Sawyer N, Nguyen T, Im DS, Stocco R, Bellefeuille JN, Abramovitz M, Cheng R (2000). Characterization of the human cysteinyl leukotriene 2 receptor. J Biol Chem.

[B11] Coleman RA, Eglen RM, Jones RL, Narumiya S, Shimizu T, Smith WL, Dahlen SE, Drazen JM, Gardiner PJ, Jackson WT (1995). Prostanoid and leukotriene receptors: a progress report from the IUPHAR working parties on classification and nomenclature. Adv Prostaglandin Thromboxane Leukot Res.

[B12] Labat C, Ortiz JL, Norel X, Gorenne I, Verley J, Abram TS, Cuthbert NJ, Tudhope SR, Norman P, Gardiner P (1992). A second cysteinyl leukotriene receptor in human lung. J Pharmacol Exp Ther.

[B13] Walch L, Norel X, Gascard JP, Brink C (1999). Cysteinyl-leukotriene receptors in pulmonary vessels. J Physiol Pharmacol.

[B14] Mita H, Hasegawa M, Saito H, Akiyama K (2001). Levels of cysteinyl leukotriene receptor mRNA in human peripheral leucocytes: significantly higher expression of cysteinyl leukotriene receptor 2 mRNA in eosinophils. Clin Exp Allergy.

[B15] Mellor EA, Frank N, Soler D, Hodge MR, Lora JM, Austen KF, Boyce JA (2003). Expression of the type 2 receptor for cysteinyl leukotrienes (CysLT2R) by human mast cells: Functional distinction from CysLT1R. Proc Natl Acad Sci USA.

[B16] Early SB, Barekzi E, Negri J, Hise K, Borish L, Steinke JW (2007). Concordant modulation of cysteinyl leukotriene receptor expression by IL-4 and IFN-gamma on peripheral immune cells. Am J Respir Cell Mol Biol.

[B17] Duroudier NP, Tulah AS, Sayers I (2009). Leukotriene pathway genetics and pharmacogenetics in allergy. Allergy.

[B18] Daniels SE, Bhattacharrya S, James A, Leaves NI, Young A, Hill MR, Faux JA, Ryan GF, le Souef PN, Lathrop GM (1996). A genome-wide search for quantitative trait loci underlying asthma. Nature.

[B19] Kimura K, Noguchi E, Shibasaki M, Arinami T, Yokouchi Y, Takeda K, Yamakawa-Kobayashi K, Matsui A, Hamaguchi H (1999). Linkage and association of atopic asthma to markers on chromosome 13 in the Japanese population. Hum Mol Genet.

[B20] Park JS, Chang HS, Park CS, Lee JH, Lee YM, Choi JH, Park HS, Kim LH, Park BL, Choi YH (2005). Association analysis of cysteinyl-leukotriene receptor 2 (CYSLTR2) polymorphisms with aspirin intolerance in asthmatics. Pharmacogenet Genomics.

[B21] Evans JF (2003). The cysteinyl leukotriene receptors. Prostaglandins Leukot Essent Fatty Acids.

[B22] Brink C, Dahlen SE, Drazen J, Evans JF, Hay DW, Nicosia S, Serhan CN, Shimizu T, Yokomizo T (2003). International Union of Pharmacology XXXVII. Nomenclature for leukotriene and lipoxin receptors. Pharmacol Rev.

[B23] Beller TC, Maekawa A, Friend DS, Austen KF, Kanaoka Y (2004). Targeted gene disruption reveals the role of the cysteinyl leukotriene 2 receptor in increased vascular permeability and in bleomycin-induced pulmonary fibrosis in mice. J Biol Chem.

[B24] Corrigan C, Mallett K, Ying S, Roberts D, Parikh A, Scadding G, Lee T (2005). Expression of the cysteinyl leukotriene receptors cysLT(1) and cysLT(2) in aspirin-sensitive and aspirin-tolerant chronic rhinosinusitis. J Allergy Clin Immunol.

[B25] Schreiber E, Matthias P, Muller MM, Schaffner W (1989). Rapid detection of octamer binding proteins with 'mini-extracts', prepared from a small number of cells. Nucleic Acids Res.

